# Assessment of Neutrophil Chemotaxis Upon G-CSF Treatment of Healthy Stem Cell Donors and in Allogeneic Transplant Recipients

**DOI:** 10.3389/fimmu.2018.01968

**Published:** 2018-09-11

**Authors:** Anna Thunström Salzer, Maria J. Niemiec, Ava Hosseinzadeh, Marios Stylianou, Fredrik Åström, Marc Röhm, Clas Ahlm, Anders Wahlin, David Ermert, Constantin F. Urban

**Affiliations:** ^1^Department of Radiation Sciences, University of Umeå, Umeå, Sweden; ^2^Department of Clinical Microbiology & Laboratory of Molecular Infection Medicine Sweden, Umeå University, Umeå, Sweden

**Keywords:** neutrophil, granulocyte colony stimulating factor (G-CSF), allogeneic transplant, chemotaxis, hematopoietic stem cell donor

## Abstract

Neutrophils are crucial for the human innate immunity and constitute the majority of leukocytes in circulation. Thus, blood neutrophil counts serve as a measure for the immune system's functionality. Hematological patients often have low neutrophil counts due to disease or chemotherapy. To increase neutrophil counts and thereby preventing infections in high-risk patients, recombinant G-CSF is widely used as adjunct therapy to stimulate the maturation of neutrophils. In addition, G-CSF is utilized to recruit stem cells (SCs) into the peripheral blood of SC donors. Still, the actual functionality of neutrophils resulting from G-CSF treatment remains insufficiently understood. We tested the *ex vivo* functionality of neutrophils isolated from blood of G-CSF-treated healthy SC donors. We quantified chemotaxis, oxidative burst, and phagocytosis before and after treatment and detected significantly reduced chemotactic activity upon G-CSF treatment. Similarly, *in vitro* treatment of previously untreated neutrophils with G-CSF led to reduced chemotactic activity. In addition, we revealed that this effect persists in the allogeneic SC recipients up to 4 weeks after neutrophil engraftment. Our data indicates that neutrophil quantity, as a sole measure of immunocompetence in high-risk patients should be considered cautiously as neutrophil functionality might be affected by the primary treatment.

## Introduction

Neutrophils are the most common leukocytes in blood and constitute 50–70% of the white blood cells corresponding to an absolute neutrophil count of 1.8-7.7 × 10^9^/l blood (1). As part of the first line of defense they immediately migrate to the site of an infection following gradients of chemoattractants released by tissue-dwelling, pathogen sensing cells (2). Like macrophages, neutrophils are professional phagocytes. However, unlike macrophages, they are loaded with preformed granules containing antimicrobial effector molecules (3). This allows neutrophils to kill invading microbes very efficiently. During phagocytosis, neutrophils engulf microbes into the phagosome that subsequently fuses with granules to form an antimicrobial environment that toxifies microorganisms by reactive oxygen species (ROS), antimicrobial peptides and proteins (4, 5). To eradicate microbes in the extracellular milieu neutrophils either degranulate antimicrobial effectors into the extracellular milieu (6) or deliver their effectors embedded into a web-like structure, known as neutrophil extracellular traps (2, 7). Clinically relevant neutropenia and severe neutropenia are defined by neutrophil counts below 1.5–2 × 10^9^/l or 0.5 × 10^9^/l, respectively (1, 8). Neutropenia occurs transiently or chronically in a variety of diseases and disorders and renders individuals more susceptible to infections (1, 9–13). A major group at risk for neutropenia are patients treated for hematological malignancies: Chemo- and radiation therapy often affect hematopoiesis, thereby reducing neutrophil production (8). Furthermore, immunosuppressive drugs such as azathioprine or mycophenolate can depress neutrophil counts (14, 15). Accordingly, persistent neutropenia is a known risk factor for invasive bacterial and fungal infections after receiving a hematopoietic stem cell (HSC) transplant (16, 17). Today, the granulocyte colony-stimulating factor (G-CSF) is widely used as a prophylactic treatment to counteract neutropenia and to prevent infections, for instance, in patients undergoing systemic chemotherapy (18, 19). However, studies on lymphoma patients revealed that G-CSF given as primary prophylaxis could not increase overall survival or reduce infection-related mortality (18). Another study on patients undergoing HSC transplantation while receiving G-CSF treatment showed a slightly decreased infection rate but no difference in treatment-related mortality (20). HSCs are transplanted either by autologous or allogeneic routes into patients suffering from different malignant and non-malignant hematopoetic disorders, for instance, acute myeloid leukemia, myeloma, or thalassemia major (8). To increase the number of pluripotent cells in peripheral blood, the donors receive G-CSF prior to the process of harvesting HSCs from peripheral blood (21). Concomitantly, the numbers of circulating neutrophils increase. Naturally, the cytokine G-CSF is a major factor controlling the development and function of neutrophils (22, 23). G-CSF regulates proliferation, maturation, survival, and functional activation of these cells (24). During the course of an infection, serum levels of G-CSF increase which in turn enhances proliferation of granulocytic precursor cells and an increased level of circulating neutrophils (23). In our study we aimed to analyze the quality of neutrophils resulting from G-CSF-treatment. We investigated key neutrophil functions in cells isolated from blood of healthy individuals recruited as HSC donors before and after G-CSF treatment as well as from allogeneic HSC recipients at different time points after receiving the HSC graft from G-CSF-treated donors. While oxidative burst and phagocytosis were unaffected in all these samples, chemotaxis was significantly reduced upon treatment with G-CSF. Strikingly, impaired chemotaxis was detectable in neutrophils from HSC recipients up to 4 weeks post engraftment.

## Materials and methods

### Ethical statement

Ethical approval (register number 09-210M, project number CFU-12/09) for the study was obtained from the Regional Ethical Review Board, Umeå, Sweden. All parts of the study were performed according to the Declaration of Helsinki. The patients were included after informed written consent.

### Subjects and treatment time line

Blood samples were collected between 2010 and 2017 at Umeå University Hospital, Sweden. The first cohort consisted of healthy individuals selected by Hematology Unit clinicians to donate for bone marrow transplantation (Table 1). For approval, SC donors underwent general health examination. The first blood sample from donors of this study was collected shortly after initial health check and before receiving the first dose of G-CSF (Figure S1A). Before donating HSCs, donors received 10 μg G-CSF/kg/day (“Nivestim™”, filgrastim, Pfizer) over 4 days to be able to donate HSCs by a continuous flow apheresis procedure. The second donor blood sample was collected on the day of HSC harvest, thus at day 5 after 4 days of G-CSF treatment, immediately before the apheresis procedure started (Figure S1A).

**Table 1:** Age and gender distribution of the cohorts: HSC donors and allogeneic transplant recipients.

	**Allogeneic transplanted (*n* = 11)**	**Stem cell donors (*n* = 12)**	***p*-value**
Demographics	Caucasian	Caucasian	NA
Mean Age (y)/ 95% percentile	49.6/ 40.2–58.9	47.0/ 37.4–56.6	0.68
Gender (m/f)	7/4	5/7	NA
Acute myeloid leukemia	*n* = 3		
Acute lymphocytic leukemia	*n* = 2		
Myelofibrosis	*n* = 2		
Myelodysplastic syndrome	*n* = 2		
Chronic myelogenous leukemia	*n* = 1		
Hemophagocytic lymphohistiocytosis	*n* = 1		

*NA, not analyzed or applying*.

The second cohort consisted of patients undergoing allogeneic HSC transplantation (Table 1). Blood samples were taken from the patients 2 weeks after successful neutrophil engraftment which was defined by absolute neutrophil count of >1.0 × 10^9^/l (Figure S1B). Depending on the individual patient, one additional sample was taken either 4 or 8 weeks post engraftment (Figure S1B). When comparing the two groups (HSC donors vs allogeneic transplant recipients) there was no statistical significant difference between the groups according to age (mean 47.0 y vs. 49.6 y, respectively) (Table 1). To control for maturity of circulating neutrophils, blood of 4 additional HSC donors (not included before in table 1) was collected *prior* and *post* G-CSF treatment and neutrophils were isolated using the same isolation procedure. In addition, we used blood from healthy volunteers not involved in any clinical treatment for *in vitro* cell stimulation analyses.

### Neutrophil isolation and maturity assessment

Peripheral venous blood was collected in EDTA-containing tubes and neutrophils were isolated using a Histopaque-1119 separation followed by a Percoll gradient, as previously described (25, 26). Viable cells were counted in a Neubauer chamber using trypan blue staining and subsequently diluted according to assay protocols. As demonstrated earlier, this procedure leads to a neutrophils purity of > 93% (27). To assess neutrophil maturity, full blood as well as isolated neutrophil fractions were analyzed for their exact cell composition. We prepared blood smears on glass slides as well as smears from neutrophil isolations, which were resuspended in plasma from the same sample. These smears were fixed and Giemsa stained. Trained personnel used these smears to perform a comprehensive blood differentiation analysis at the Unit of Laboratory Medicine at the Norrland University Hospital in Umeå (project number FFLA01). All samples were analyzed in a blinded fashion. Band cells were considered immature, cells containing a segmented nucleus were considered mature (28, 29).

### Media and incubation

If not stated otherwise, neutrophil functionality assays were performed in RPMI medium without phenol-red (Lonza) and incubation occurred under standard cell culture conditions: 37°C, 5% CO_2_, humid atmosphere.

### Chemotaxis

Neutrophils chemotaxis was quantified using a transwell setup similar to previous description (30, 31). Briefly, 5 × 10^6^ neutrophils/ml were stained with 3.3 μM bis-2-carboxyethyl-5-[and-6]-carboxyfluorescein-AM (Sigma-Aldrich) for 20 min at room temperature, washed, and resuspended in medium with 0.05% human serum albumin. Fluorescence-impermeable transwell inserts (BD Falcon, HTS FluoroBlok, 3 μm pore size, PET membrane) were loaded with 5 × 10^5^ cells each and placed into wells of 24-well plates containing 600 μl RPMI, with or without 10 nM formyl-methionyl-leucyl-phenylalanine (fMLF). Fluorescence intensity (FI) with excitation 485 nm and emission 520 nm was detected in a plate reader (FLUOstar OMEGA; BMG Labtech) with 5% CO_2_ and 37°C over the course of 30 min. Medium only served as blank and 5 × 10^5^ stained cells seeded directly into the lower compartment served as 100% FI reference. If indicated, we added 10 ng/ml or 50 ng/ml G-CSF (human recombinant G-CSF, Sigma-Aldrich) to the respective neutrophil sample. Subsequently, cells were washed in medium and used as described. Thus, if not stated otherwise, neutrophils are unstimulated prior to the analysis. Migration was calculated as percent of 100% reference Migration of unstimulated cells was subtracted from the fMLF-induced migration. Half migration time was determined as time required for 50% of maximum migration. Velocity at half migration time was calculated as Δ%/min (see Figure S2).

### Reactive oxygen species production

Oxidative burst was quantified by luminol-based chemiluminescence (26). Briefly, 5 × 10^4^ neutrophils per well were seeded in white 96-well plates with 50 μM luminol and 1.2 U/ml horseradish peroxidase (both Sigma-Aldrich). Neutrophils were stimulated with 100 nM phorbol 12-myristate 13-acetate (PMA) or left untreated. Chemiluminescence as a measure of hydrogen peroxide production was detected every 2 min for 3 h in an Infinite 200 luminometer (Tecan). ROS were quantified by the area under the curve (AUC) calculated for the time given (3 h) and reduced by background of unstimulated cells.

### Phagocytosis

Phagocytosis was assessed using pH rodo beads (pHrodoTM Red *S. aureus* bioparticles conjugated for phagocytosis, Lifetechnologies) that change their FI pH-dependently. Briefly, 5 × 10^4^ neutrophils per well were added into a black 96-well plate with 100 μg beads/well. Beads put directly into a buffer with pH 4 served as 100% FI control. Beads in medium with pH 7.4 served as blank. Neutrophils pre-treated with 10 μM cytochalasin D for 15 min to block phagocytosis served as negative control. FI (540/580 nm) was measured every 60 min for 3 h (FLUOstar Omega plate reader, BMG Labtech).

### Plasma cytokine quantification

Along with neutrophil isolation from HSC donors, 1.5 ml of plasma were collected and immediately stored at−80°C. These samples were used to analyze levels of human G-CSF and interleukin 8 (IL-8) using Luminex ELISA assays (Bio-Rad, Bio-Tech, respectively). Assays were performed according to manufacturer's protocol using a magnetic beads washer. For G-CSF quantification, the samples (Luminex, Bio-Rad) were diluted 1:4 in sample diluent. For IL-8 quantification in plasma from HSC donors, a high-sensitive Luminex assay (BioTech) was used with standard range 0.78–3200 pg/ml. Samples were diluted 1:2 in calibrator diluent RD6-40 and added to plates for subsequent analysis with a Bio-Plex reader (Bio-Plex 200 system, Bio-Rad). Plasma samples were used for the analysis of C-reactive protein (CRP) which was quantified using the CRPL3 cobas® assay (Roche Diagnostics Scandinavia) performed by the Clinical Chemistry Laboratory at Umeå University Hospital according to the manufacturer's recommendations and the standard ISO/IEC 17025:2005 with continuous evaluations by the Swedish Board for Accreditation and Conformity Assessment.

### *In vitro* cytokine release

To analyze IL-8 release upon G-CSF stimulation, neutrophils were isolated from healthy volunteers, and 5 × 10^5^ neutrophils were treated with 50 ng/ml G-CSF. After 30 min, 1 μg/ml LPS was added as a co-stimulant for 6 h. As control, cells were treated with medium only. After 6 h, supernatants were collected and directly frozen at −80°C. The IL-8 concentration was quantified according to manufacturer's protocol (BioLegend Human IL-8 ELISA Max standard set).

### Quantification of cell surface fMLP receptor

G-CSF treated as well as untreated neutrophils were stained with labeled monoclonal anti-fMLP receptor antibody coupled to PE (PE, MACS, Miltenyi Biotec) in PBS supplemented with 3% FCS in a final dilution of 1:10 and incubated for 30 min at 8°C. Cells were washed 3 times in PBS with 3% FCS followed by flow cytometry analysis (BD Biosciences LSR II). The population of neutrophils were identified using FSC-A and SSC-A. At least 10^4^ cells were analyzed per sample and all samples were run in duplicates.

### Statistics

We used Graph-Pad Prism V 5.04 for statistical analysis. Data was analyzed using Students *t*-test, and one-way ANOVA with Tukey's multiple comparison tests. Mann-Whitney U-, Wilcoxon signed-rank and Kruskal-Wallis tests were used when data was not normally distributed. A *p*-value below 0.05 was considered significant.

## Results

### G-CSF treatment in healthy HSC donors reduces neutrophil *ex vivo* chemotaxis

**Figure 1:**
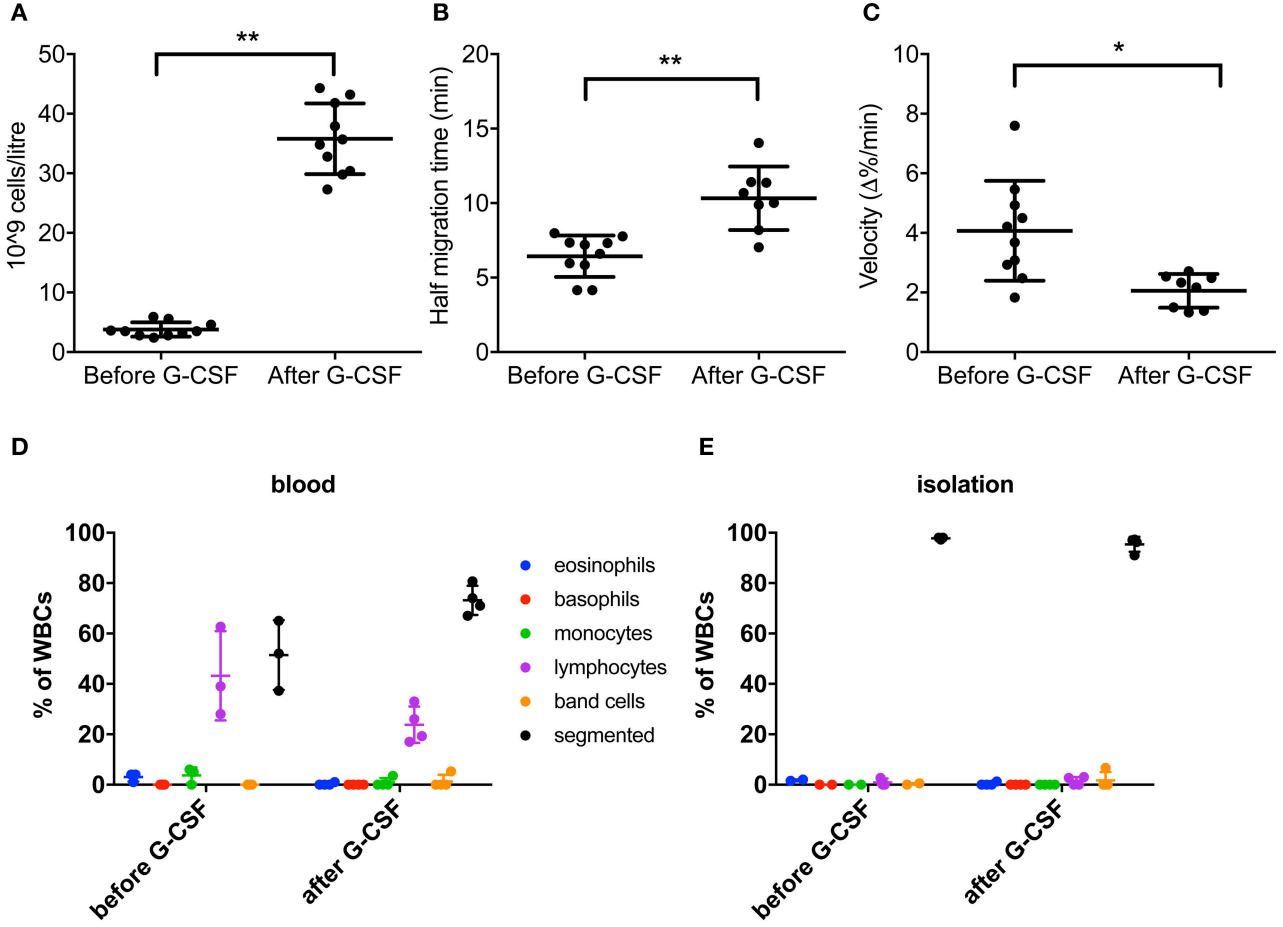
G-CSF treatment affects neutrophil migration. Stem cell donors before and after treatment were analyzed for neutrophil function. **(A)** Neutrophil count measured before and after G-CSF-treatment in the stem cell donor group (*n* = 10). Revealed a significant increase in the number of circulating neutrophils after G-CSF treatment. **(B)** Distribution of half migration time during chemotaxis within the stem cell donor group before and after G-CSF-treatment. Half migration time was defined as the time required for half-maximal fluorescence signal indicative for migration toward 10 nM fMLP. **(C)** The distribution of velocity during chemotaxis before and after G-CSF-treatment is indicated. Velocity was measured as Δ %/min at half migration time indicating the speed of the neutrophils when they have reached half maximal fluorescence in the bottom well. Blood cell differentiation analysis **(D)** before and **(E)** after purification of neutrophils from stem cell donors before (*n* = 3) and after (*n* = 4) GCSF treatment confirmed that the purification enriched mature neutrophils >95% independent of previous G-CSF treatment. Statistical analysis was performed using a Wilcoxon signed-rank test **(A–C)**. ^*^p < 0.05, ^**^p < 0.01.

We determined chemotaxis of neutrophils isolated from peripheral blood of G-CSF-treated HSC donors (Figure S1). As expected, the total neutrophil count increased in the blood of these individuals ~ 9-fold upon treatment (Figure 1A). When analyzing the neutrophils *ex vivo*, we detected that neutrophils isolated from HSC donors after G-CSF-treatment displayed significantly increased half migration time and a reduced velocity at half migration time compared to neutrophils before treatment (Figures 1B,C). In contrast, spontaneous, unstimulated migration of neutrophils was similar using neutrophils from donors before and after G-CSF treatment (Figure S2) as determined by half migration time and velocity at half migration time (Figure S3). In summary, chemotaxis of neutrophils from HSC donors after G-CSF-treatment was reduced compared to neutrophils from the same individuals before treatment. This suggests that G-CSF administration in healthy HSC donors could reduce the ability of circulating neutrophils to migrate upon chemotactic stimulation. Of note, the number of immature neutrophils, so-called band cells, was negligibly small below 2% before and after G-CSF treatment in both, full blood and neutrophil isolation fractions (Figures 1D,E) as determined by a complete blood differentiation analysis. Further, the neutrophil isolation strategy chosen for this study proved to be efficient in enriching the neutrophil content to > 95%.

**Figure 2:**
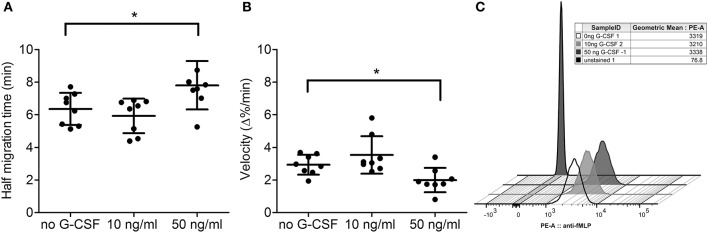
Systemic G-CSF treatment impairs chemotaxis in neutrophils from healthy donors. Purified neutrophils from peripheral blood of healthy, untreated donors (*n* = 8) were incubated with 0, 10, or 50 ng/ml human recombinant G-CSF. Thereafter, a chemotaxis assay was performed using fMLP as chemoattractant and half migration time **(A)** as well as velocity at half migration time **(B)** was determined. Neutrophils pre-treated with 0, 10, and 50 ng/ml of G-CSF respectively were stained with a PE-labeled anti-fMLP receptor antibody. The histogram is showing a representative experiment of three similar replications **(C)**. Statistical analysis was performed with a Kruskal-Wallis test for **(A)** (*p* = 0.0098) and **(B)** (*p* = 0.0093), complemented with Mann-Whitney U-tests showing that samples 0 and 50 ng/ml are significantly different for **(A,B)** with ^*^p < 0.05.

We next investigated whether G-CSF had a similar effect on neutrophil chemotaxis if directly applied to neutrophils *in vitro*. For this purpose, isolated neutrophils from peripheral blood of eight healthy volunteers were pre-incubated with 10 and 50 ng/ml G-CSF *prior* to the migration assay. Indeed, we observed that high concentrations of G-CSF decreased the chemotactic ability of isolated neutrophils (Figures 2A,B). With 50 ng/ml G-CSF, chemotaxis was significantly impaired as determined by half migration time and velocity at half migration time. This demonstrates that G-CSF can directly decrease migration activity of isolated neutrophils *ex vivo* although to a smaller extent. To test whether this effect could result from a removal of the fMLF-specific G-protein coupled receptor, we quantified its surface expression by flow cytometry. We incubated purified neutrophils with 10 and 50 ng/ml G-CSF and quantified surface exposure of the receptor by staining with antibodies directed against fMLF receptor (Figure 2C). We could not observe any differences between treated and untreated neutrophils suggesting that surface exposure of the fMLF receptor on neutrophils was not affected by G-CSF treatment.

### G-CSF administration to healthy HSC donors alters levels of IL-8 and CRP in plasma

**Figure 3:**
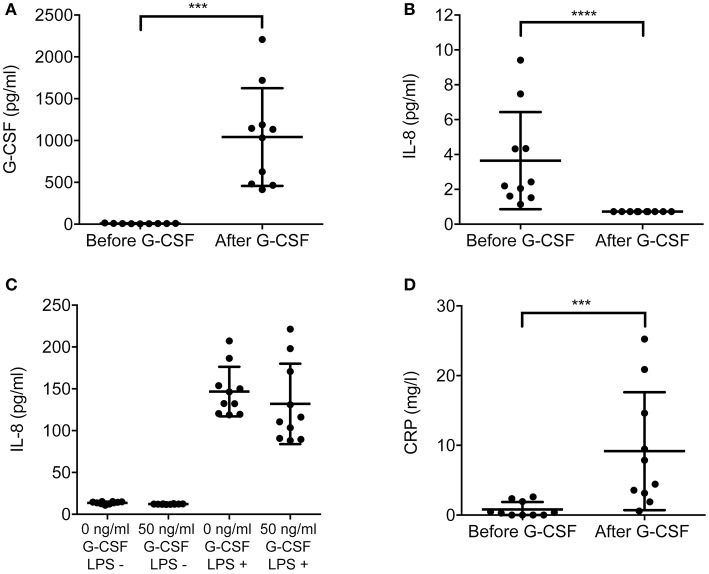
Plasma from G-CSF-treated individuals contains lower amount of IL-8 than untreated controls. G-CSF **(A)**, IL-8 **(B)**, and CRP **(D)** concentrations in plasma obtained from stem cell donors (*n* = 10) before and after G-CSF-treatment. Each value was determined from a mean of four technical replicates for G-CSF and IL-8. For IL-8 **(B)** all the samples after G-CSF treatment were below the range of the assay (0.72 pg/ml). To be able to perform statistical analysis the values were extrapolated to 0.72 pg/ml. **(C)** Levels of IL-8 secreted by neutrophils *in vitro* after G-CSF (50 ng/ml) or mock treatment for 30 min and subsequently either stimulated with LPS (1 μg/ml, labeled as LPS +) or left untreated (labeled as LPS -). The levels of IL-8 were measured by ELISA after 6 h stimulation. The CRP values **(D)** were measured as single measurements for each donor before and after G-CSF treatment. Statistical analysis was performed using a paired students *t*-test **(C)** and Mann-Whitney U-test **(A**,**B**,**D)** when data was not normally distributed. ^***^p < 0.001 and ^****^p < 0.0001.

Next, we investigated potential mechanisms for the reduced chemotaxis of G-CSF-treated neutrophils. Interleukin 8 (IL-8) is a central chemokine for neutrophil recruitment contributing to the extravasation of neutrophils to the site of infection (32). C-reactive protein (CRP), an acute phase serum protein of hepatic origin, has been previously implicated to suppress neutrophil chemotaxis *in vitro* (33). We therefore, analyzed plasma samples from HSC donors, taken before and after G-CSF treatment, for IL-8 and CRP levels. To reveal possible correlations, we also quantified plasma levels of G-CSF. After treatment, G-CSF concentrations were significantly increased, 1042 pg/ml compared to 8 pg/ml before treatment (Figure 3A). Notably, we observed a significant decrease of IL-8 in plasma of G-CSF-treated individuals as compared to the samples taken before treatment (Figure 3B). After treatment, IL-8 levels were at detection limit (0.72 pg/ml), whereas before treatment all obtained values for IL-8 were within detection range. Despite this observation in plasma, *in vitro* priming of neutrophils from healthy volunteers with 50 μg/ml G-CSF did not affect the release of IL-8 upon stimulation with endotoxin lipopolysaccharide from *Escherichia coli* (Figure 3C). In contrast to IL-8, acute phase protein CRP levels in plasma significantly increased upon G-CSF treatment (Figure 3D).

### Neutrophil chemotaxis remains reduced in allogeneic transplant recipients

**Figure 4:**
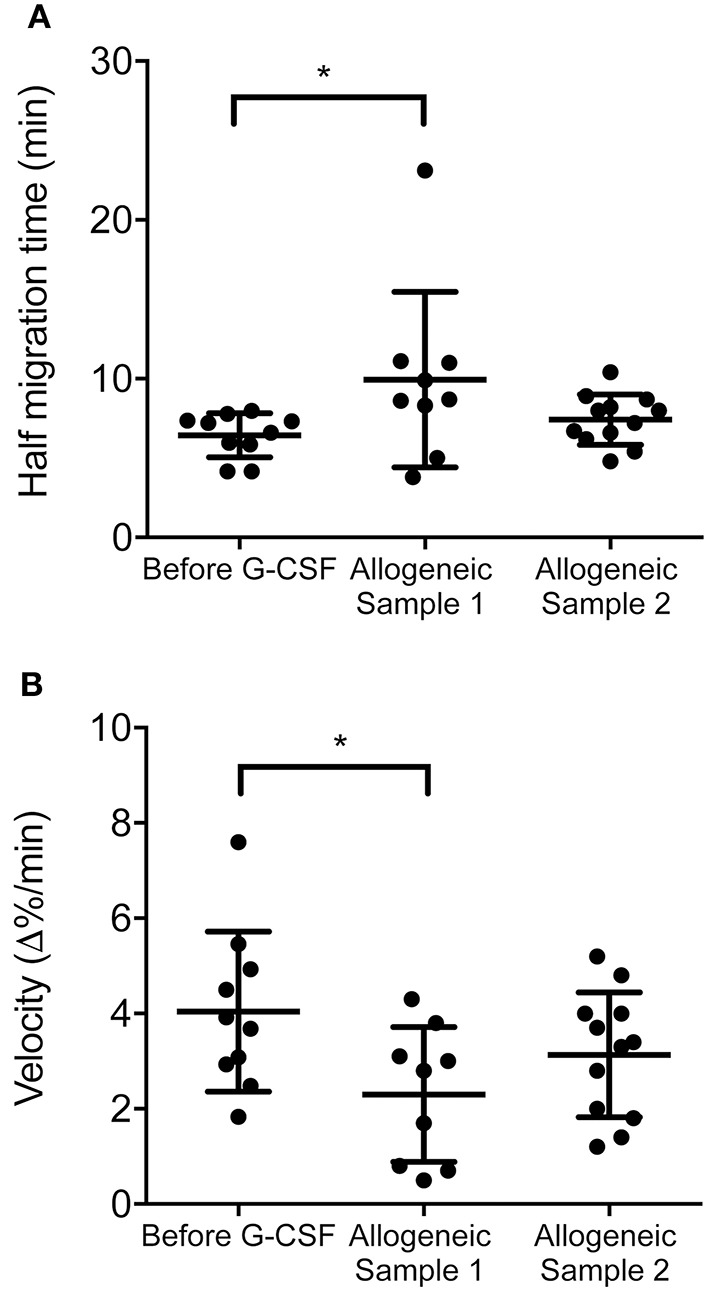
The chemotactic activity remains impaired in allogeneic transplant recipients. **(A)** Distribution of half migration time in the stem cell donors before G-CSF treatment (*n* = 10), serving as controls, and samples from patients undergoing allogeneic stem cell transplantation at a medium of 48.9 days (*n* = 9), indicated as sample 1, and 80.2 days (*n* = 12), indicated as sample 2, after transplant, respectively. The half migration time is measured as the time it takes to reach half of max fluorescent and is a measure of neutrophil speed in the process of chemotaxis. **(B)** The distribution of velocity during chemotaxis comparing stem cell donors before G-CSF with samples from patients undergoing allogeneic stem cell transplantation as described above. The evaluation shows that the velocity at half max migration goes down early after allogeneic stem cell transplantation which confirms that the chemotaxis in neutrophils is slower early after stem cell transplantation. Statistical analysis was performed with a Kruskal–Wallis test for **(A)** (*p* = 0.0466) and **(B)** (*p* = complemented with Mann–Whitney U-tests showing that samples “before G-CSF treatment” and “allogeneic sample 1” are significantly different for **(A,B)** with ^*^p < 0.05.

We compared neutrophil chemotaxis from controls and allogeneic transplant recipients 2 weeks after neutrophil engraftment (Figure S1) and observed significant differences. Half migration time is increased (Figure 4A) and velocity at half migration time is decreased for the group of allogeneic transplant recipients (Figure 4B) similarly to the G-CSF-treated healthy HSC donors (Figures 1B,C). This indicates that the G-CSF-mediated effects on neutrophil chemotaxis remained in the recipient 2 weeks after neutrophil engraftment. Healthy HSC donors prior to G-CSF treatment serving as controls were homogenous for age, but not for gender when compared to the transplant recipients. However, evaluating our data in a gender-separated fashion we do not find differences between the sexes (Figure S4). This indicates that our findings do not correlate with differences in gender or age between the groups.

Analysis of neutrophils sampled 4 weeks after neutrophil engraftment and beyond (Figure S1) showed that this effect was transient. Neutrophil chemotaxis was not significantly different from those of the untreated controls (Figures 4A,B). Thus, our data implies that there might be a window of increased risk of infection for the transplant recipients after engraftment (30 days in average) in which neutrophil numbers are high, but they might still have functional limitations (Table 1).

### ROS production and phagocytosis unaffected by G-CSF treatment

**Figure 5:**
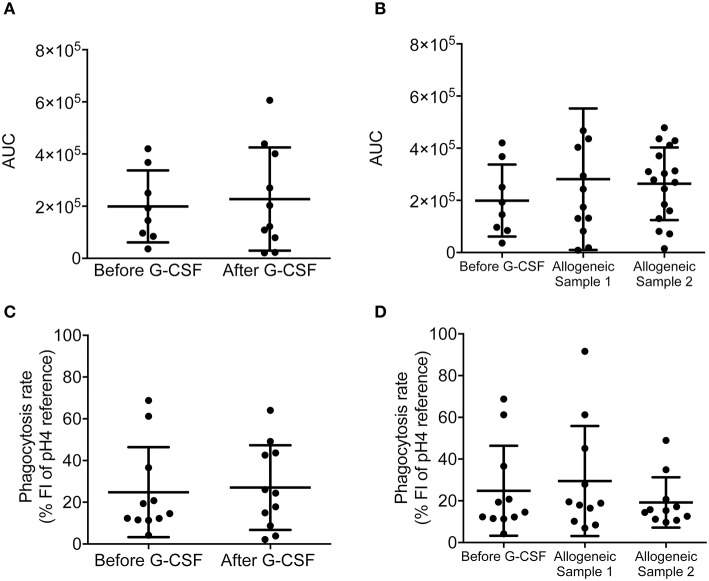
ROS production and phagocytosis are not affected by G-CSF in stem cell donors and neither in allogeneic transplant recipients. **(A)** Production of ROS by neutrophils was measured as area under the curve (AUC) in the stem cell donor group before and after G-CSF-treatment (*n* = 10). Statistical analysis was performed using an unpaired students *t*-test. **(B)** Production of ROS measured as AUC in the samples from patients undergoing allogeneic stem cell transplantation using the samples from the stem cell donors before G-CSF-treatment as a control. Statistical analysis was performed using a Kruskal-Wallis test due to skewness of data. **(C,D)** Phagocytosis measured as the uptake of pH-sensitive pHrodo beads that triggered phagocytosis by neutrophil isolated from **(C)** stem cell donor group before and after G-CSF-treatment (*n* = 10) and from **(D)** patients undergoing allogeneic stem cell transplantation using the samples from the stem cell donors before G-CSF-treatment as a control. Phagocytosis rate was calculated as percent out of 100 percent control. Statistical analysis was performed using a One-way ANOVA with Tukey's multiple comparison tests. Statistical analyses in **(A-D)** did not reveal significance *p* < 0.05.

ROS, produced by the NADPH oxidase complex in phagocytes, are important mediators of cell signaling and very powerful effectors of antimicrobial killing. Thus, we investigated whether G-CSF treatment alters total ROS production in neutrophils stimulated with PMA for 3 h. We could not detect a difference in ROS production by neutrophils from HSC donors before and after G-CSF-treatment (Figure 5A). Similarly, neutrophils from allogeneic transplant recipients did not display a significantly altered ROS production when comparing sample 1 and sample 2 (Figure 5B). This implies that the neutrophil's potential to produce ROS was unaffected by G-CSF treatment.

As professional phagocytes, engulfment of microbes is a key mode of action of neutrophils. We therefore quantified phagocytic uptake using fluorescently-labeled beads tagged with surface molecules from *Staphylococcus aureus*. Upon engulfment, these beads increase fluorescence intensity in the acidic milieu of the mature phagosome. To confirm actual phagocytosis, we included neutrophils treated with cytochalasin D as a negative control. Cytochalasin D inhibits cytoskeleton rearrangements and thus allows discrimination between adherence and phagocytosis. When comparing the phagocytic uptake by neutrophils from HSC donors before and after G-CSF treatment as well as the neutrophils from the allogeneic transplant recipients at different time points of harvest we could not observe any significant difference (Figures 5C,D). Of note, we observed that neutrophils from G-CSF-treated individuals could engulf beads even in the presence of the cytoskeleton inhibitor cytochalasin D, while phagocytic uptake of neutrophils from HSC donors *prior* to G-CSF treatment was blocked as expected (Figure S5). This may indicate that G-CSF renders neutrophils less susceptible against cytochalasin D. Notably, pH of surrounding medium was regularly controlled and did not change during the course of the experiments. In conclusion, ROS production and phagocytosis were not affected by G-CSF treatment.

## Discussion

Using G-CSF to mobilize HSCs has revolutionized the treatment of blood malignancies as it often replaces the classical bone marrow transplant (34). Instead of harvesting bone marrow surgically from the pelvis bone, which requires, e.g., general anesthesia, the cells of interest are collected from the peripheral blood. Yet again, there is growing evidence that G-CSF treatment alters more physiological functions than the blood cell count (22, 35). Our study analyzed key neutrophil functions, i.e., chemotaxis, ROS production, and phagocytosis, in isolated neutrophils from the peripheral blood of HSC donors and allogeneic transplant recipients by using well-established *in vitro* stimuli and assays. The individuals included in our study received standard dosages of G-CSF (10 μg/kg/d) and the resulting plasma concentrations were in the expected ranges (34, 35). As expected, G-CSF treatment substantially increased the number of neutrophils in the circulation. When analyzing the functionality of these neutrophils, we revealed that if derived from healthy HSC donors treated with G-CSF, the cells showed reduced *ex vivo* chemotaxis toward fMLF. Other key functions tested, i.e., ROS production and phagocytosis, seemed to remain unaltered. Strikingly, the chemotactic impairment observed in neutrophils from HSC donors persisted in the group of allogeneic transplant recipients. Even after transplantation and novel granulopoiesis within the transplant recipient, newly emerging neutrophils showed the same migration defect as the neutrophils from the HSC donors. Of note, the recipients themselves received no prophylactic G-CSF. According to our data, it took several weeks until normal neutrophil activity was regained. Notably, we observed the chemotactic impairment even though the allogeneic transplant recipient group was heterogeneously composed with diversity regarding the underlying disease. Nevertheless, these patients frequently receive immunosuppressive therapy, such as steroids or calcineurin inhibitors, which in turn were shown to impair neutrophil functions. However, the effects of these type of drugs are usually pleiotropic and do not specifically target neutrophil chemotaxis. For instance, calcineurin inhibitors can impair chemokinesis of neutrophils on vitronectin (36), reduce oxidative burst induced by fMLF (37) and diminish phagocytic activity (38). Further, the content of immature neutrophils was <2% in both, full blood and neutrophil isolation fractions—even after G-CSF treatment.

Our results confirm previous studies describing impaired neutrophil functionality in allogeneic transplant recipients. Sosa et al. reported that neutrophil chemotaxis is impaired up to 4 months after HSC transplantation (39) and Katoh et al. observed a decrease in chemotactic activity for up to 12 months (40). Azzara et al. even pointed toward a reduced motility of neutrophils from chemotherapy patients treated with G-CSF (41). Still, these studies were performed with neutrophils from cancer patients while we also included otherwise healthy HSC donors yet observed similar abnormalities. This implies that G-CSF treatment in general affects neutrophils and is supported by Kerst et al. who described phenotypically and functionally altered neutrophils upon G-CSF treatment in healthy donors (42). Few studies addressed the impact of G-CSF administration on neutrophils in healthy individuals, in our case HSC donors. Dale and coworkers investigated the combined impact of G-CSF and corticosteroids on neutrophil function (43), whereas Höglund and colleagues similar to our study investigated the singular effect of G-CSF (44). The authors observed a reduced migratory activity of neutrophils after several doses of G-CSF and the transient nature of this effect, as it disappeared several weeks after treatment. Our study confirms and extends on these findings, since we directly combine investigations of healthy HSC donors with allogeneic transplant recipients as well as linking our observations to CRP and IL-8 levels and direct *in vitro* effects as discussed in more detail below. Interestingly, *in vitro* studies triggering neutrophils directly with G-CSF observed a rapid stimulation of random mobility that declined within about half an hour (45). In our experiments showing reduced mobility due to G-CSF treatment, neutrophils were incubated with G-CSF for that same amount of time and subsequently challenged with fMLF to induce active movement. Taken together, these observations suggest a possible “exhaustion” of chemotactic potential due to random mobility if neutrophils are directly challenged with G-CSF. Notably, in our study, reduced *in vitro* chemotaxis did unlikely result from decreased surface exposure of the fMLF receptor.

More strikingly, we demonstrate that reduced neutrophil chemotaxis persisted within the allogeneic transplant recipients at 2 weeks post HSC engraftment. The average life span of neutrophils in circulation is relatively short; it is estimated to be not longer than a few days (46–48). So, in these patients, the neutrophils analyzed did not stem from the first round of newly generated cells, but more likely from a following generation of neutrophils. Our findings are in good agreement with a study investigating neutrophil functions in autologous bone marrow transplantation recipients (49). Humphreys et al. report decreased chemotaxis of neutrophils upon G-CSF treatment, which persisted until 2 weeks post engraftment (49). Yet again, the effects observed were detected for neutrophils derived from HSCs from patients with a hematological disorder, and not, like in our case, from healthy HSC donors.

In addition to reduced chemotaxis, we show that IL-8 plasma levels of HSC donors are reduced compared to untreated controls after G-CSF treatment. That decrease is contradictory to a study by Watanabe et al. where the authors reported elevated levels of IL-8 after G-CSF-treatment even though the G-CSF treatment regimen was virtually identical to our donors (50). Moreover, this study showed a correlation between the IL-8 levels and the amount of harvested CD34^+^ cells that are used as a measure for HSC mobilization. Again, this is in contrast with our findings: We could not detect any correlation between IL-8 levels and amount of harvested CD34+ cells or the neutrophil count in circulation. Since IL-8 is pivotal for neutrophil chemotaxis, we conclude that the significant reduction of IL-8 plasma levels might contribute also to reduced chemotaxis of neutrophils. However, it remains unclear, which cells are responsible for IL-8 production under these conditions.

Neutrophils are crucial for the innate immunity. G-CSF is therefore used in neutropenic patients as a preventive treatment to avoid infections. Still, a study with transplant recipients reported that preventive G-CSF treatment did not alter infection-related mortality in these patients (51). While this supports the relevance of reduced neutrophil functionality, i.e., the ability to reach the site of infection *in vivo* by chemotaxis, others described an unchanged infection risk in G-CSF-treated HSC donors (52). Taken together, this suggests a potential compensation by number if neutrophil functionality is reduced.

Previously, it was shown that CRP levels increased upon G-CSF treatment (53). Our data confirms this finding. Moreover, our observations provide a possible link between increased CRP plasma levels in G-CSF-treated individuals and a negative effect on neutrophil chemotaxis. Of note, CRP reduced the chemotactic ability of neutrophils *in vitro* (33).

To judge whether reduced chemotaxis in neutrophils is a direct effect of the G-CSF present in blood, we have detected G-CSF plasma levels. Even in treated HSC donors, G-CSF plasma levels ranged around 1 ng/ml. Other studies have described slightly higher plasma concentrations ranging from 4 to > 15 ng/ml (35). In our *in vitro* experiments with purified neutrophils, 10 ng/ml G-CSF had no effect on neutrophil chemotaxis, only 50 ng/ml G-CSF impacted neutrophil chemotaxis. Taking this into consideration, our findings indicate that lower IL-8 and higher CRP concentrations in combination with a direct effect of G-CSF might render neutrophils less motile in G-CSF-treated individuals. Reduced IL-8 levels in plasma of G-CSF treated individuals could stem from non-neutrophil IL-8-sequestering cells.

In our cohorts, ROS production and phagocytosis were not affected by systemic G-CSF treatment in healthy HSC donors or in allogeneic recipients. Oxidative burst and phagocytosis remained unchanged in all cohorts and at all time points of sampling. This specificity suggests that the reduced chemotaxis in our cohorts is not a result of general immaturity of neutrophils mediated by G-CSF as immaturity of neutrophils is usually accompanied by the impairment of other functions as well (54, 55). It should be mentioned that other studies investigating neutrophil function of allogeneic recipients as reviewed by Ramaprasad et al. reported effects on ROS production or phagocytosis (56). Scholl and coworkers, for instance, observed an impaired oxidative burst in neutrophils from allogeneic recipients when stimulating with *E. coli*, but not when stimulating with PMA, confirming our findings (57). Macey and colleagues described impaired phagocytic ability of neutrophils after transplantation, however these patients received additional G-CSF treatment, which is in contrast to our study (58).

Allogeneic transplant recipients are susceptible to infections (59). With a weakened immune system, often combined with immunosuppressive treatments, an infection can often be a fatal complication. To judge the immune status of patients at risk, it is still common practice to rely on the neutrophil count in blood. To the best of our knowledge, there is no clinical tool available to evaluate the functionality of innate immune cells. In our study, we show that neutrophils recruited by G-CSF treatment in healthy HSC donors displayed decreased functionality in chemotaxis. Notably, this decrease persisted temporarily in allogeneic transplant recipients after neutrophil engraftment—even though these patients did not receive G-CSF themselves. Therefore, we conclude that it is possible that G-CSF could have a similar effect on neutrophils in blood cancer patients undergoing G-CSF treatment. Our study provides evidence that this regimen might require reconsideration that is more thorough. Henceforth, our findings imply that chemotaxis is a sensitive neutrophil function, which seems to be impaired early on during treatment and disease. From another point of view, our results provide evidence that chemotaxis could serve as reporter for neutrophil functionality in the clinical setting. With this, our study serves as a promising starting point for future prospective studies on chemotaxis as functional marker related to infection risk in patients with hematological diseases and other patient groups receiving G-CSF as prophylactic treatment.

## Author contributions

DE, AW, CA, MN, and CU designed the research study. AW, FÅ, and AT recruited and took care of the donors and patients. AT, MN, AH, DE, MS, and MR performed the experiments. AT, MN, AH, MS, and CU analyzed the data. AT, MN, DE, and CU wrote the paper.

### Conflict of interest statement

The authors declare that the research was conducted in the absence of any commercial or financial relationships that could be construed as a potential conflict of interest.
