# Supplementary figures and images for: Assessment of Neutrophil Chemotaxis Upon G-CSF Treatment of Healthy Stem Cell Donors and in Allogeneic Transplant Recipients
Source: Front Immunol. 2018 Sep 11;9:1968. doi: 10.3389/fimmu.2018.01968 (PMC6141688; doi:10.3389/fimmu.2018.01968)

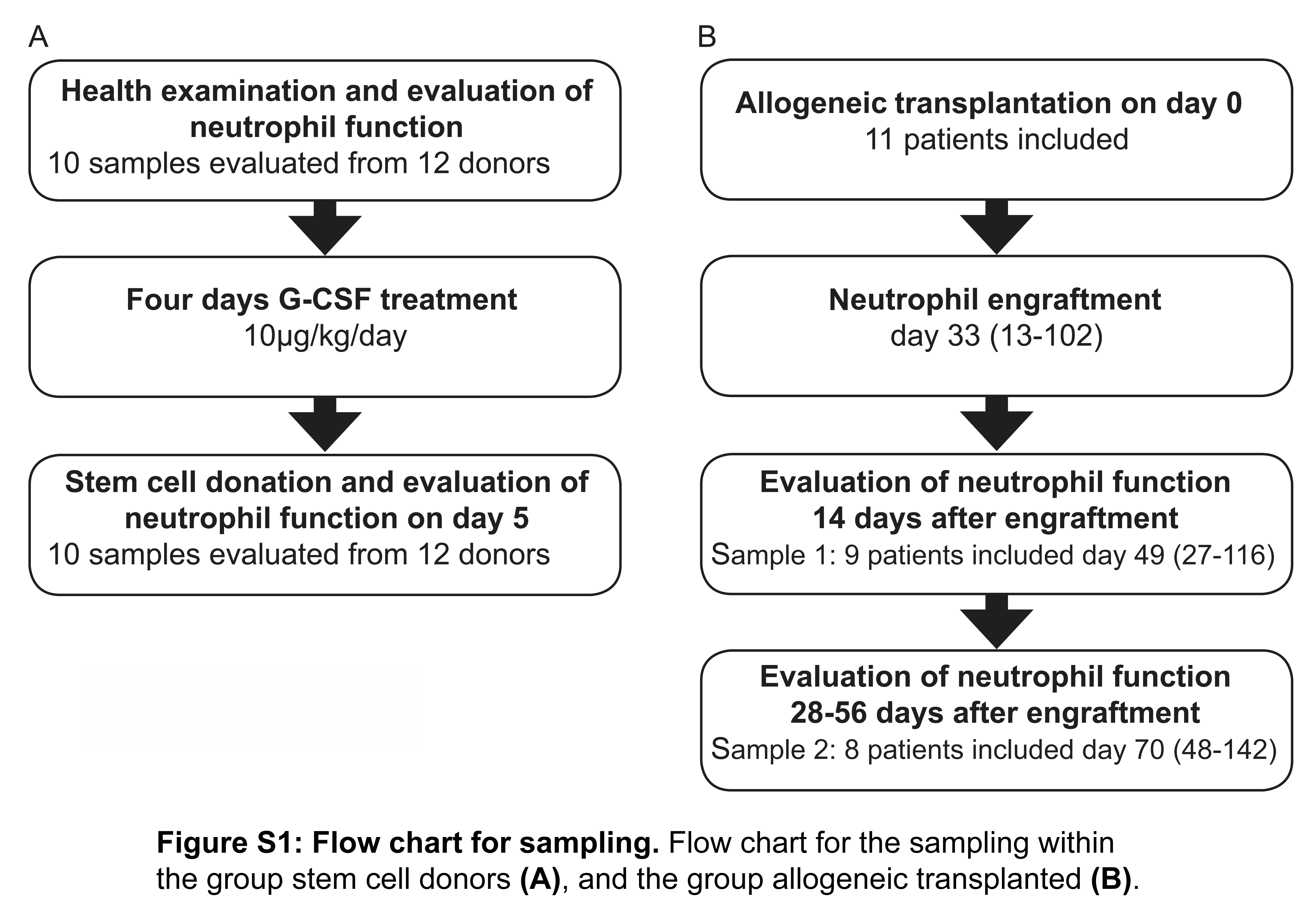

Supplement: Supplementary file 1 [file Image_1.tif]

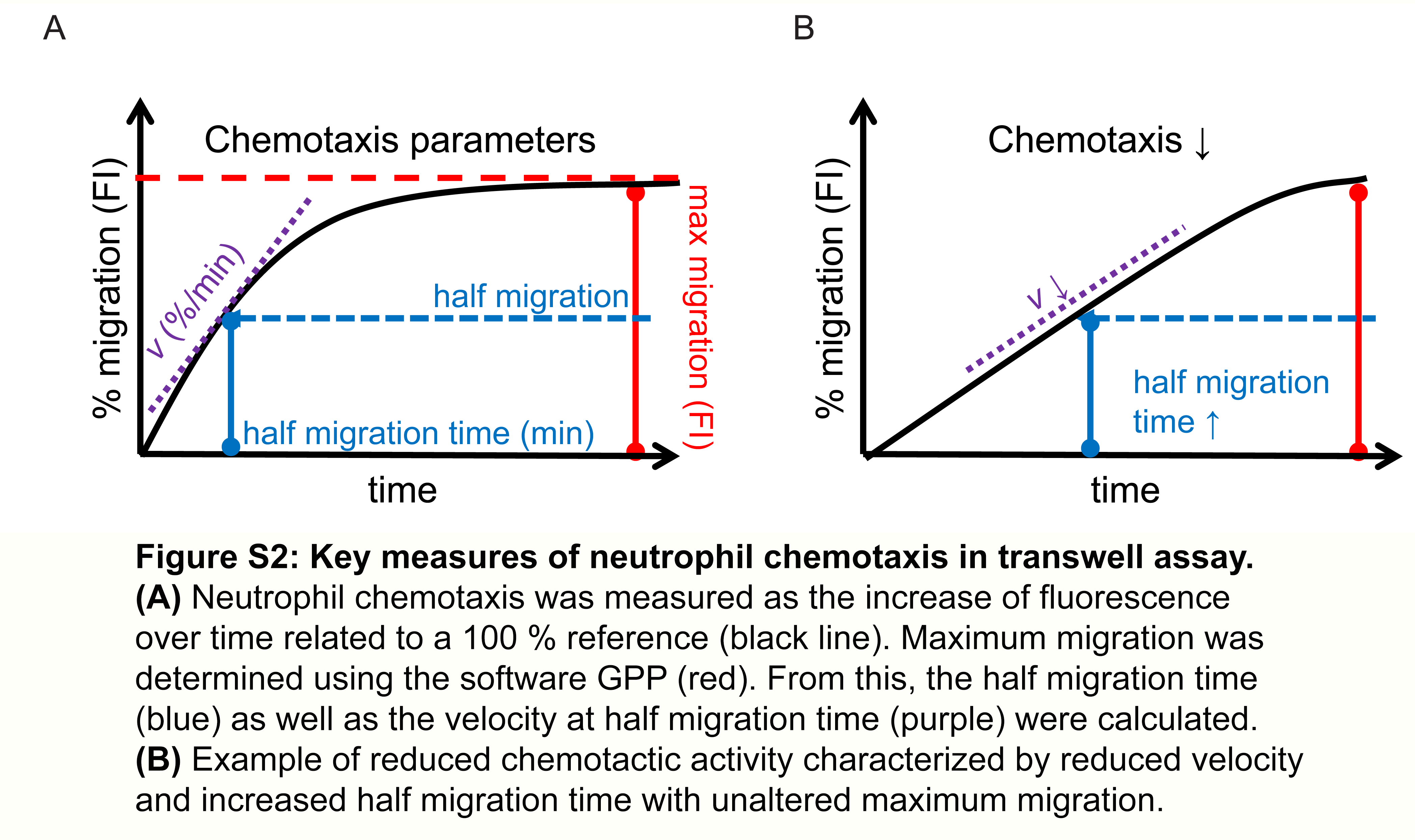

Supplement: Supplementary file 2 [file Image_2.tif]

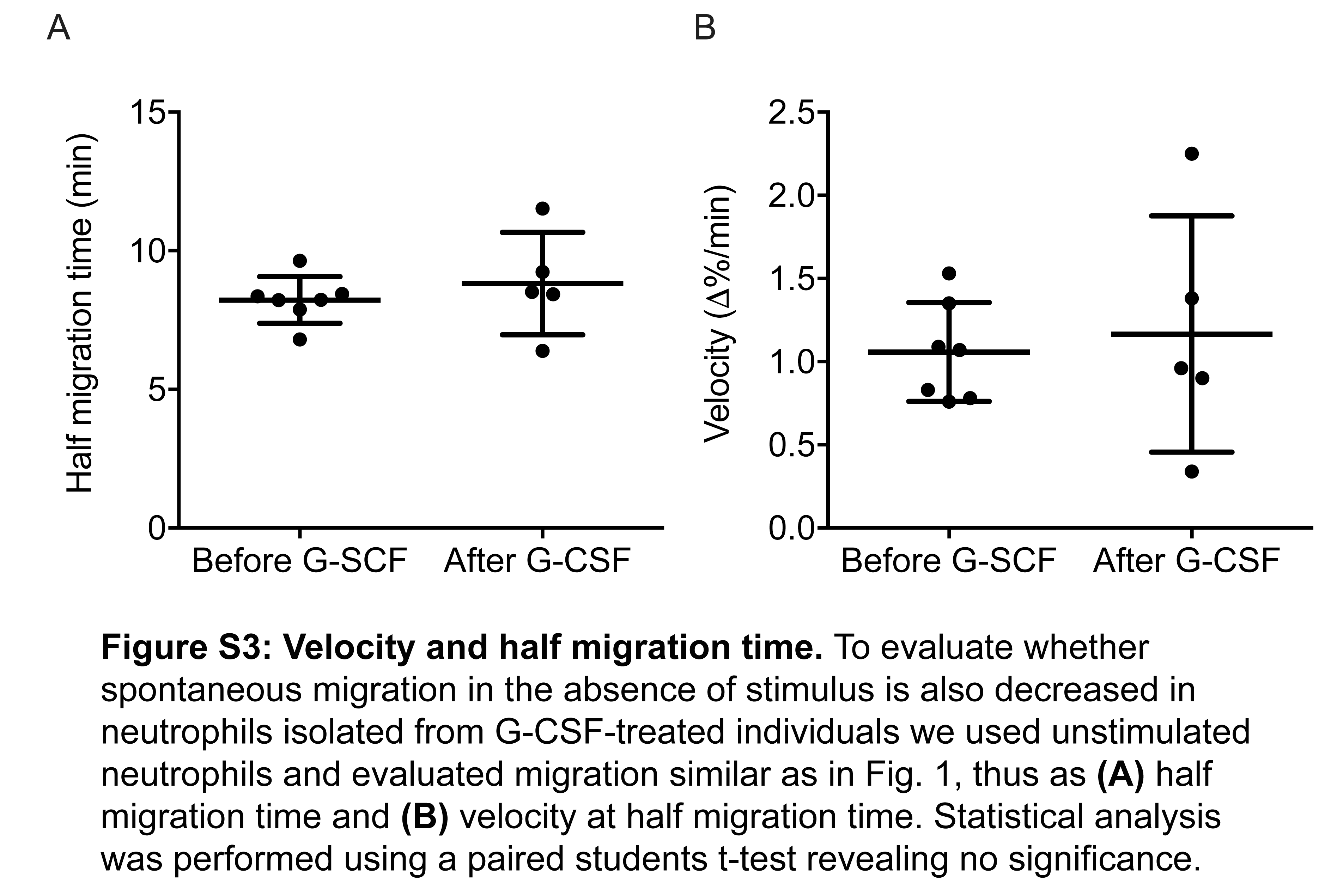

Supplement: Supplementary file 3 [file Image_3.TIF]

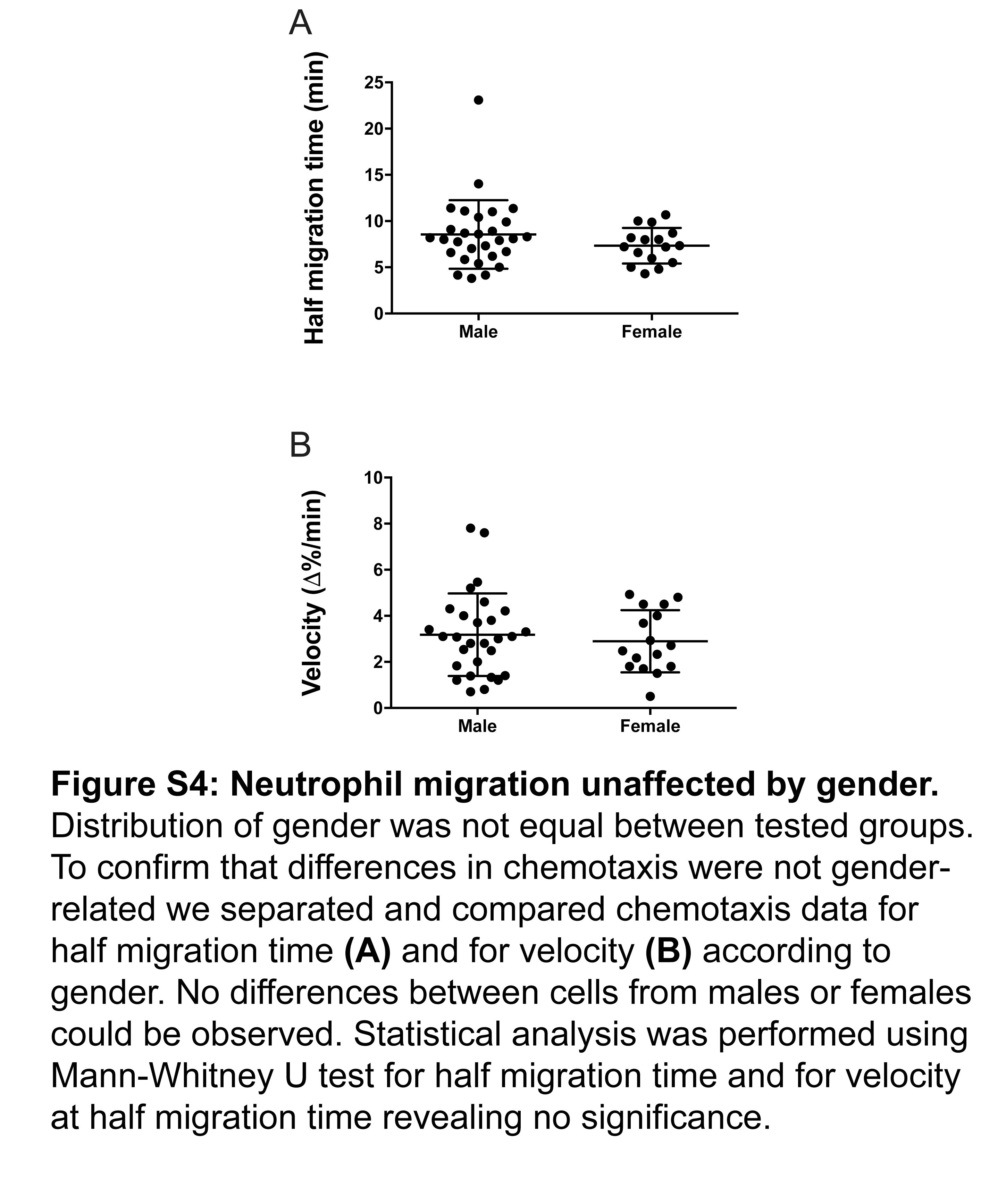

Supplement: Supplementary file 4 [file Image_4.TIF]

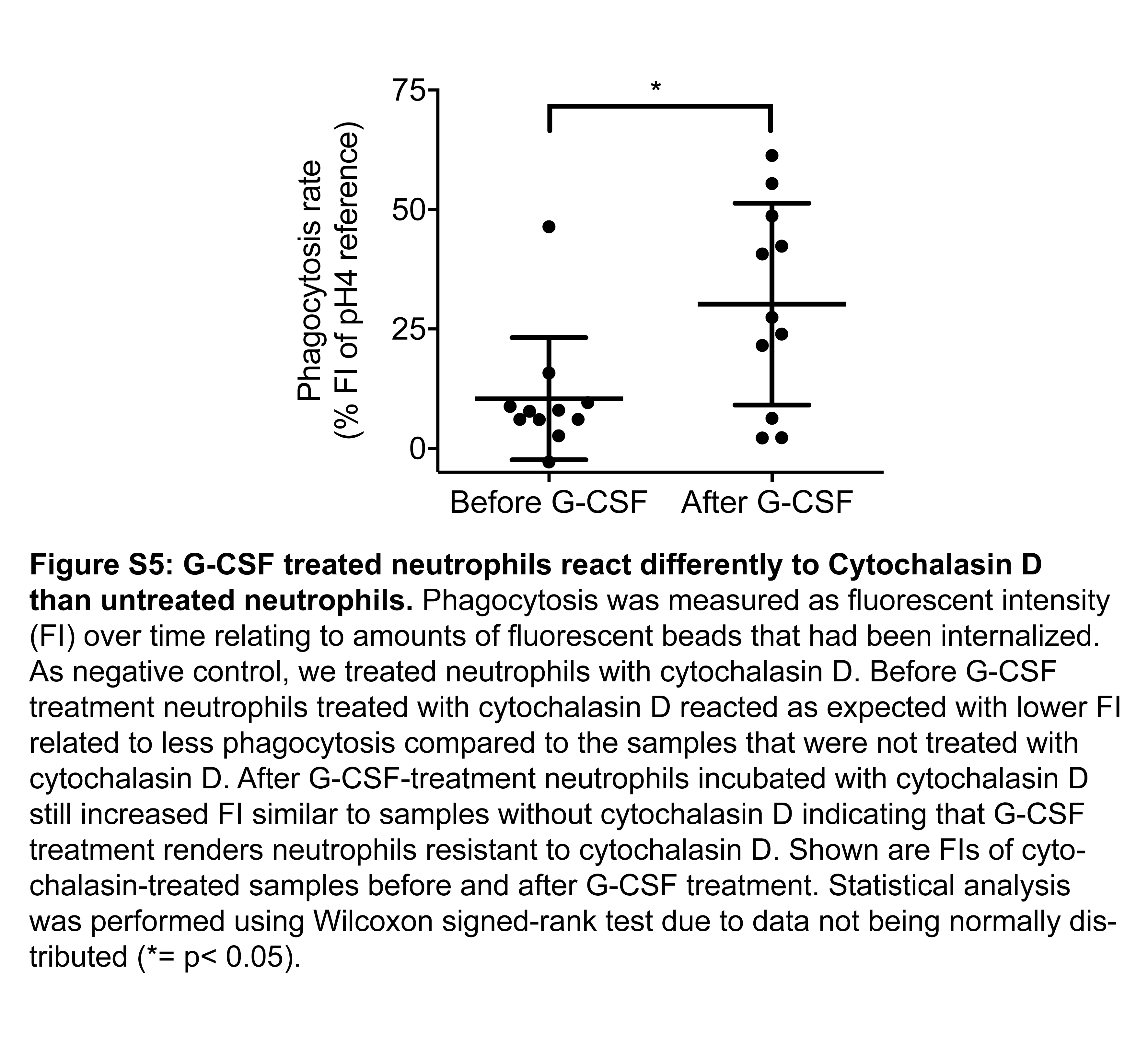

Supplement: Supplementary file 5 [file Image_5.tif]
